# Effect of Rumen-Protected Cod Liver Oil Supplementation on Fatty Acid Profile of Meat from Limousin and Red Angus Cattle

**DOI:** 10.3390/ani15131856

**Published:** 2025-06-23

**Authors:** Andrzej Zachwieja, Ewa Pecka-Kiełb, Anna Zielak-Steciwko, Bożena Króliczewska, Jowita Kaszuba, Robert Kupczyński

**Affiliations:** 1 Animal Breeding, Wroclaw University of Environmental and Life Sciences, ul. Chelmonskiego 38C, 51-631 Wroclaw, Poland; andrzej.zachwieja@upwr.edu.pl (A.Z.); anna.zielak-steciwko@upwr.edu.pl (A.Z.-S.); jowita.kaszuba@upwr.edu.pl (J.K.); 2 Department of Biostructure and Animal Physiology, Wroclaw University of Environmental and Life Sciences, Norwida Str. 31, 50-375 Wroclaw, Poland; bozena.kroliczewska@upwr.edu.pl; 3 Department of Environment Hygiene and Animal Welfare, Wroclaw University of Environmental and Life Sciences, ul. Chelmonskiego 38C, 51-631 Wroclaw, Poland; robert.kupczynski@upwr.edu.pl

**Keywords:** oil supplementation, fatty acid, fat, protein, meat, Limousin and Red Angus bulls

## Abstract

**Simple Summary:**

Meat is one of the main dietary sources of essential fatty acids, which the human body cannot synthesize. Beef, particularly its intramuscular fat, contains a relatively high amount of unsaturated fatty acids, which may positively influence human health. As consumer awareness of food quality grows, the meat industry is shifting toward the production of functional foods. One practical approach is to modify the fatty acid composition of meat through feeding strategies, which are more efficient than long-term genetic approaches. In this study, Limousin and Red Angus beef cattle were fed rumen-protected cod liver oil. The supplementation resulted in a decreased content of myristic acid (C14:0) and stearic acid (C18:0) in Limousin meet, saturated fatty acids associated with negative health effects. Both breeds also showed increased levels of cis-oleic acid in meat and fat, along with higher overall concentrations of monounsaturated fatty acids (MUFAs) in adipose tissue. These changes in fatty acid profile are considered beneficial. Reducing the dietary intake of C14:0 and C18:0, while increasing with MUFA consumption has been linked to improved cardiovascular function and may aid in managing diet-related chronic diseases. This study demonstrates that targeted nutritional interventions can effectively enhance the health value of beef.

**Abstract:**

In beef cattle production, both diet and breed are key factors influencing the composition and quality of meat. This study evaluated the effects of rumen-protected cod liver oil supplementation on meat and fat quality parameters in Limousin (*n* = 30) and Red Angus (*n* = 30) bulls maintained under identical conditions. During the final three weeks of finishing, animals received 100/g/day of cod liver oil. Red Angus bulls exhibited a significantly higher intramuscular fat content in meat compared to Limousin (*p* < 0.01). The study demonstrated a reduction (*p* < 0.05) in intramuscular fat content in both breeds receiving cod liver oil supplementation. In Limousin, cis-oleic acid (C18:1 cis-9) levels increased significantly in meat (*p* < 0.05) without a concurrent increase in trans isomers. Subcutaneous fat in both breeds showed a significant increase in monounsaturated fatty acids in the supplemented group compared to the control (*p* < 0.01). Limousin bulls also showed reduced levels of myristic acid (C14:0) and stearic acid (C18:0) in subcutaneous fat. Additionally, Limousin muscle tissue showed significantly higher (*p* < 0.01) concentrations of C18:3n3, C22:6n3, and total polyunsaturated fatty acids (PUFAs) compared to Red Angus. These finding indicate that the short-term dietary inclusion of rumen-protected cod liver oil in cattle rations enhances the nutritional profile of beef, potentially offering benefits for human health.

## 1. Introduction

The growing number of studies on functional foods with targeted effects on the human body reflects increasing consumer interest in functional products [1,2,3,4,5]. In addition to health benefits, consumers are giving a greater emphasis on the sensory qualities of food, such as taste [6]. In this context, beef plays an important role, as the fatty acid composition of muscle and adipose of beef tissue not only determines the nutritional value of meat, but also affects its technological and sensory quality, including shelf life [7].

Due to the established link between dietary saturated fats and cardiovascular diseases of human, health authorities worldwide recommend reducing the intake of saturated fatty acids (SFAs) and cholesterol while encouraging the consumption of polyunsaturated fatty acids (PUFAs) [8]. In 2003, the World Health Organization (WHO) recommended that, to reduce the risk of coronary heart disease, the ratio of polyunsaturated to saturated fatty acids (PUFAs/SFAs) in food should exceed 0.45 (WHO, 2003) [9]. In 2023, the WHO called for the global elimination of industrially produced trans fatty acids from the food supply and recommended minimizing their intake in the human diet (WHO, 2023) [10]. Intramuscular fat in beef is characterized by a relatively high proportion of unsaturated fatty acids, which may have a positive impact on human health [6,11]. Furthermore, these unsaturated fatty acids enhance sensory characteristics such as the aroma, flavor, juiciness, and tenderness of meat [12,13].

In cattle, both genetic and non-genetic factors influence the content and composition of subcutaneous and intramuscular fat [13,14]. Differences in intramuscular fatty acid profiles have been observed among various breeds, including Angus, Hereford, and Charolais [15]. Similar changes in fatty acid profiles have also been observed in Limousin and Aberdeen Angus cattle, which are very popular among breeders [16,17,18]. Additional variations have been reported in crossbreeds such as Angus–Simmental, Wagyu–Simmental, and Chinese–Simmental [19]. But breeders often choose Red Angus and Limousin cattle due to their ease of calving, efficient feed utilization, and rapid adaptation to environmental conditions [17]. In addition, both breeds are characterized by high meat quality and a flavor that is desirable to consumers [20]. However, the effects of rumen-protected cod liver oil supplementation on fatty acid deposition in beef muscle and fat across different breeds remain underexplored. Although the concentration of desirable compounds in meat can be improved through genetic selection or crossbreeding, such approaches are time-consuming and costly. Nutritional strategies, including the use of functional feed additives, offer a more immediate and cost-effective method of enhancing meat quality [21,22,23,24,25].

One promising nutritional intervention involves marine-derived products, particularly fish oil. Fishing and the subsequent processing of fish intended for human consumption generate large quantities of by-products, including heads, viscera, skins, tails, scales, minced meat, and blood [26]. It is estimated that only 50–60% of the wet mass of fish obtained from marine fisheries is used for direct human consumption, while the remaining 40–50% is processed into fish oil and fishmeal [27,28]. According to the Marine Ingredients Organization (IFFO), which represents the global marine ingredients sector, by-products accounted for approximately 54% of global fish oil production in 2023 [29]. Cod offal, containing approximately 4.3% lipids, is considered a suitable raw material for fish oil production [30]. Fish oil is a valuable source of long-chain polyunsaturated fatty acids that support animal health, improve reproductive performance, and contribute to enhanced average daily weight gain. These fatty acids also aid in digestive efficiency and the absorption of fat-soluble vitamins [31,32]. However, the economic feasibility of fish oil use varies widely across regions. In countries such as Norway or Iceland, where cod liver oil is a by-product of the fishing industry, its inclusion in ruminant diets may be more economically viable. Supplementation with marine-derived products such as fish oil or marine algae has been shown to positively affect the concentration of polyunsaturated fatty acids (PUFAs) in beef [33,34,35], and enhance the levels of conjugated linoleic acid (CLA) in meat and blood plasma [35]. However, when unsaturated fatty acids are administered in an unprotected form, they are susceptible to microbial hydrogenation in the rumen, which can reduce fiber digestibility and dry matter intake in ruminants [36]. To circumvent ruminal biohydrogenation and maintain the integrity of supplemented fatty acids, rumen-protected forms of fat are used [37,38]. This strategy allows for unsaturated fatty acids to bypass the rumen and be absorbed in the small intestine, thereby preserving their biological value [39,40]. Rumen protection prevents the microbial conversion of unsaturated fatty acids into their saturated forms, thus increasing the nutritional and functional quality of the resulting meat [41]. The use of fish oil in cattle diets is associated with both physiological benefits and additional production costs. Although the production of premium meat products typically requires greater financial investment, consumers are increasingly willing to accept higher prices due to greater awareness of the relationship between nutrition and human health [42]. Given the costs associated with animal feed, it is reasonable to assess changes in the nutritional composition of meat following the short-term inclusion of lipid-based supplements in ruminant diets. Marino et al. (2019) [43] reported that flaxseed supplementation for 40 days prior to slaughter enhances the concentration of n-3 fatty acids and decreases the n-6/n-3 ratio in meat, despite flaxseed containing unprotected fatty acids. Similarly, Hennessy et al. [44] observed an increase in plasma fatty acid methyl ester (FAME) concentrations after just 10 days of rumen-protected n-3 fatty acid supplementation. Short-duration supplementation protocols have also been explored with other compounds, such as magnesium, which is typically administered 7 to 14 days pre-slaughter to improve carcass quality and reduce pre-slaughter stress [45]. Therefore, this study aimed to evaluate the effects of short-term supplementation with rumen-protected cod liver oil on the fat and protein content and fatty acid profile in the muscle and adipose tissue of Limousin and Red Angus bulls.

## 2. Materials and Methods

The procedures were carried out in accordance with European regulations (Directive 2010/63/EU), and all efforts were made to minimize animal suffering and stress. In accordance with relevant legislation, the collection of material post-mortem during routine animal slaughter does not require approval from the Ethics Committee.

### 2.1. Animal Experimental Design

**Figure 1:**
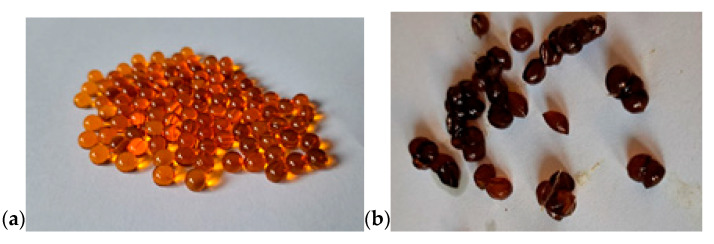
(**a**) Microcapsules before in vitro incubation. (**b**) Microcapsules after 24 h incubation. No visible damage was observed; rupture occurred only under mechanical pressure. A change in capsule coloration was noted due to the action of rumen fluid.

The study was carried out on a commercial beef cattle herd located in the West Pomeranian Voivodeship, Poland. The herd comprised 350 Limousin and 500 Red Angus animals. The experimental groups comprised young beef cattle fattened to a body weight of 500–550 kg. The animals were kept in group pens housing 20–30 individuals each, in accordance with established animal welfare standards. None of the animals showed any signs of disease throughout the study period. Throughout the study, the health and performance of all animals were regularly monitored. All animals remained healthy and continued to meet the inclusion criteria for the duration of the experimental period. The experiment followed a randomized block design, and animals were assigned based on breed, age, and body weight. A total of 60 finishing bulls (Limousin *n* = 30; Red Angus *n* = 30) were selected and grouped into four blocks based on age (18 ± 0.5 months) and body weight (680 ± 23 kg; 625 ± 21 kg, respectively). Within each block, bulls were randomly allocated to one of four treatment groups: Limousin bulls fed a control diet without added fat (CL *n* = 15); Limousin bulls supplemented with microencapsulated cod liver oil (EL *n* = 15); Red Angus bulls on a control diet (CA *n* = 15); and Red Angus bulls receiving microencapsulated cod liver oil (EA *n* = 15). All animals had unrestricted access to fresh water and were fed ad libitum using a total mixed ration (TMR) system under intensive production conditions. The feed rations were formulated in accordance with INRA standards [46]. The basal diet consisted of 80% corn silage, 15% grass silage, and 5% barley straw, supplemented with a complete feed mixture provided at 3 kg/animal/day. The nutritional composition of the feed dose is presented in Table 1. The experimental factor involved the administration of microencapsulated cod liver oil, delivered at a dose of 100 g/animal/day during the final three weeks of the fattening period. From the total daily amount of 3 kg of concentrate per animal, 0.5 kg was set aside for manual mixing with the 100 g of the experimental microcapsules and was administrated to the animals daily between 06:00 and 07:00 a.m. during routine feeding. On average, 2–5% of the roughage feed remained uneaten each day. Each microcapsule contained 50 µL of cod liver oil and was encapsulated in a gelatin matrix composed of fish-derived gelatin, glycerol, and water. The capsules were approximately spherical in shape, with a diameter of about 5 mm and a wall thickness of 0.3 mm. The fatty acid composition of the cod liver oil is shown in Table 2. Prior to in vivo administration, in vitro stability tests were conducted. Using the Ankom rumen simulation system (Ankom Technology, Macedon, NY, USA), a 12 h incubation in simulated rumen fluid revealed no visible degradation of the microcapsules (Figure 1a,b). Furthermore, no significant differences were observed in the profile of major short-chain fatty acids (acetic, propionic, and butyric acids) compared to the control, indicating minimal ruminal disruption.

After the completion of the experimental period, all bulls were humanely slaughtered on the same day at a commercial, EU-approved slaughterhouse, in accordance with European Union Regulation ((EC) no. 1099/2009 [47]) on the protection of animals at the time of killing. Post-mortem, samples of the longissimus dorsi muscle were collected from the left half-carcass at the level of the 12th thoracic vertebra, along with adjacent subcutaneous fat tissue. For analysis, 10 g samples were obtained from the central portion of the muscle or fat tissue. Samples were collected prior to the meat aging process, specifically within 4–6 h post-slaughter. Efforts were made throughout the study to ensure sample uniformity. After collection, all samples were properly labeled, vacuum-sealed, and transported to the laboratory under refrigerated conditions at a constant temperature of 4 °C.

### 2.2. Chemical Analysis

Samples of roughage and the complete feed mixture were subjected to proximate chemical analysis, with all procedures conducted in accordance with the official methods of the Association of Official Analytical Chemists [48] (AOAC, 2005). Dry matter (DM) content was determined using the oven drying method (934.01), while crude protein (CP) content was assessed using the Kjeldahl method (984.13) with a FOSS Tecator 2300 Kjeltec Analyzer Unit (FOSS Tecator, Höganäs, AB, Sweden). Crude protein was calculated as total nitrogen (N) × 6.25. Ether extract (EE) was determined according to method 920.39 using the Fibertec extraction system (FOSS Tecator, Höganäs, Sweden). Neutral detergent fiber (NDF) and acid detergent fiber (ADF) contents were analyzed following method 973.18, also employing the Fibertec system [48]. The chemical composition of the feedstuffs used in the dietary rations is presented in Table 1. The fatty acid profile of the cod liver oil was determined using a method analogous to the ether extract procedure and is presented in Table 2.

### 2.3. Meat and Fat

In the collected longissimus dorsi muscle samples, fat content was determined according to the PN-ISO 1444:2000 [49] standard using a Büchi B-811 automatic extraction system. Total protein content was measured using the Kjeldahl method in accordance with the PN-75/A-04018 [50] standard, employing a DK 6 digestion unit and a UDK 129 steam distillation apparatus (VELP Scientifica, Italy).

The fatty acid profile was analyzed in both muscle and subcutaneous fat samples. Lipids from the muscle tissue were extracted using the Folch method [51] (Christie & William, 1973). Fatty acid methyl esters (FAMEs) were then prepared from both the extracted intramuscular fat and directly collected subcutaneous fat samples using the method described by Christopherson and Glass (1969) [52], which involved transesterification with 2 M KOH in methanol. The fatty acid profile of the obtained samples was determined using an Agilent Technologies 7890A gas chromatograph (Santa Clara, CA, USA) equipped with a flame ionization detector (FID). The detection range for saturated fatty acids was from C14 to C20, and for unsaturated fatty acids from C14 to C22. The chromatographic separation was performed using an HP-88 capillary column (Agilent Technologies; 100 m length, 0.25 mm internal diameter, 0.20 μm film thickness). The oven temperature was initially set at 50 °C and increased at a rate of 3 °C/min to a final temperature of 220 °C. The injector and detector temperatures were maintained at −270 °C and 270 °C, respectively. Helium was used as the carrier gas, with hydrogen, synthetic air, and nitrogen supplied to the detector. Identification of the obtained fatty acid peaks was performed by comparing the retention times to those of fatty acid methyl ester standards SupelcoTM 37 (Sigma Aldrich, St Louis, MO, USA) and CLA cis-9, trans-11 and trans-10, cis-12 (Larodan, Malmö, Sweden), using ChemStation software (Agilent Technologies). The concentration of each fatty acid was expressed in g/100 g, with 100 g representing the sum of all identified FAME peak areas.

The obtained fatty acid results from muscle and fat were used to calculate the atherogenic index (AI) and thrombogenic index (TI) according to Ulbricht and Southgate (1991) [53]:

AI = [(4 × C14:0) + C16:0]/[(ΣPUFA) + (ΣMUFA)]

TI = [C14:0 + C16:0 + C18:0]/[(0.5 × ΣMUFA) + (0.5 × n-6) + (3 × n-3) + (n-3/n-6)]

### 2.4. Statistical Analysis

Data obtained from the analysis of muscle and fat tissues were subjected to a one-way analysis of variance (ANOVA) using the STATISTICA software package, version 13.3 (StatSoft, Tulsa, OK, USA) [54]. Prior to the analysis, the data were tested for normality using the Shapiro–Wilk test and for homogeneity of variances using Levene’s test. Differences between groups were evaluated using Duncan’s multiple-range test, with statistical significance set at *p* < 0.05 and highly significant differences at *p* < 0.01.

## 3. Results

### 3.1. Chemical Composition of Meat: Protein Content, Fat Content, and Fatty Acid Profile

In Limousin cattle, the control group (CL) exhibited a significantly lower (*p* < 0.01) protein content, and a higher (*p* < 0.05) intramuscular fat content compared to the group supplemented with fish oil (EL) (Table 3). Similarly, in Red Angus cattle, a significant reduction in fat content (*p* < 0.05) was observed in the experimental group (EA) relative to the control group (CA). Overall, Red Angus meat was characterized by a significantly higher (*p* < 0.01) fat content compared to Limousin meat. In contrast, protein content was higher in groups CA, EA, and EL compared to the CL group.

The supplementation administered during the final three weeks of the fattening period influenced the concentration of saturated fatty acids (SFAs) in the longissimus dorsi muscle of Limousin cattle (Table 4). In the group receiving cod liver oil (EL), a reduction was observed in both pentadecanoic acid (C15:0) and total SFA content compared to the control group (CL).

Meat from Red Angus cattle was characterized by higher concentrations of myristic acid (C14:0), palmitic acid (C16:0), and total SFAs relative to Limousin meat. Regardless of dietary treatment, Limousin meat exhibited significantly higher levels (*p* < 0.05) of eicosanoic acid (C20:0) compared to Red Angus meat. Additionally, the proportion of C15:0 acid was significantly higher (*p* < 0.01) in the CL group compared to all other groups.

The experimental factor exerted breed-dependent effects on the proportion of individual unsaturated fatty acids in the *longissimus dorsi* muscle (Table 5). In Limousin cattle from the EL group, a significantly higher proportion (*p* < 0.05) of cis-Δ8-octadecenoic acid (C18:1n8c) and an increased n-6/n-3 fatty acid ratio were observed compared to the CL group. In Red Angus cattle, the EA group exhibited a significantly higher thrombogenic index (TI) (*p* < 0.01) and a greater total content of monounsaturated fatty acids (MUFAs) (*p* < 0.05) relative to the CA group. Additionally, the levels of α-linolenic acid (C18:3n3) and arachidonic acid (C20:4n6) were significantly lower (*p* < 0.05) in the EA group compared to CA. Breed had a notable influence on the unsaturated fatty acid profile. Red Angus meat was characterized by a higher MUFA content, as well as elevated atherogenic index (AI) and thrombogenic index (TI) values, in comparison to Limousin meat. Conversely, meat from Limousin cattle exhibited higher concentrations and a more favorable profile of polyunsaturated fatty acids (PUFAs), along with improved PUFAs/∑SFAs. In the meat of Limousin bulls fed with protected oil supplementation, higher levels of n-3 and n-6 fatty acids were observed (*p* < 0.01), along with an increased n-6/n-3 ratio (*p* < 0.05). In contrast, the meat of Red Angus bulls in the EA group showed a reduced (*p* < 0.05) level of n-3 fatty acids compared to the CA group.

### 3.2. Fatty Acid Composition of Subcutaneous Fat

The dietary supplement influenced the SFA profile of subcutaneous fat in both cattle breeds (Table 6). In the experimental groups, a significant reduction (*p* < 0.05) in heptadecanoic acid (C17:0) and stearic acid (C18:0), as well as a decrease in total SFA content (*p* < 0.01) was observed compared to the respective control groups. In addition, within the Limousin breed, the proportion of myristic acid (C14:0) was significantly lower (*p* < 0.05) in the CL group compared to the EL group. When comparing breeds, subcutaneous fat from Red Angus cattle contained higher concentrations of myristic acid (C14:0), palmitic acid (C16:0), and short-chain fatty acids (SCFAs), and lower levels of heptadecanoic acid (C17:0), stearic acid (C18:0), and eicosanoic acid (C20:0), compared to Limousin cattle.

Changes in the proportions of individual unsaturated fatty acids (UFAs) in the subcutaneous fat of beef cattle were observed in response to the applied dietary supplementation (Table 7). In both breeds, the supplement led to a significant increase (*p* < 0.05) in cis-Δ8-octadecenoic acid (C18:1n8c) and total MUFAs, as well as a significant increase (*p* < 0.01) in total UFA content compared to the respective control groups. In the experimental groups, the TI index was significantly reduced (*p* < 0.05) in both breeds. In Limousin cattle, the AI index was significantly higher (*p* < 0.05) in the CL group compared to the EL group. In the Red Angus breed, subcutaneous fat from group EA showed significantly higher levels of heptadecenoic acid (C17:1; *p* < 0.05) and cis-Δ9-octadecenoic acid (C18:1n9c; *p* < 0.01) compared to group CA. In Limousin cattle, a higher level of palmitoleic acid (C16:1) (*p* < 0.05) was recorded in group EL compared to CL. No statistically significant breed-related differences were observed in the overall profile of unsaturated fatty acids in subcutaneous adipose tissue. Moreover, the addition of cod liver oil had no effect on the total levels of n-3 and n-6 fatty acids, or their ratio in subcutaneous fat.

## 4. Discussion

In the present study, Limousin and Red Angus bulls were fattened under identical housing and feeding conditions on the same farm. This experimental design enabled a direct comparison of the effects of a protected cod liver oil supplement on selected meat and fat quality parameters in two distinct beef cattle breeds. Both Limousin and Red Angus breeds have long been favored by European cattle breeders and beef producers due to their desirable growth performance and meat quality traits [16,17].

### 4.1. Protein and Fat Content in Muscle Tissue

Available literature data suggest breed-specific differences in fat deposition, while protein levels tend to remain relatively stable across breeds [55,56]. Limousin cattle, in particular, are recognized for producing lean beef with a relatively high protein content [56], and the results of our own study are consistent with these literature findings.

To date, there are no published reports specifically evaluating the effects of protected cod liver oil supplementation in cattle diets on fat and protein content in beef. However, various studies have demonstrated that the inclusion of plant-based oils rich in unsaturated fatty acids in ruminant diets can influence the composition of animal-derived products. For instance, rapeseed oil supplementation in dairy cows has been reported to have no significant effect on meat fat content [57], though it was associated with reduced milk fat production and increased milk protein content [58]. Similarly, Oliveira et al. (2012) [59] observed that dietary supplementation with soybean and linseed oils in Nellore bulls increased muscle protein content while reducing fat deposition in the longissimus dorsi muscle. A comparable fat-reducing effect was noted in goats, where the addition of soybean and rapeseed oils decreased kidney fat accumulation [60]. In the present study, the inclusion of protected cod liver oil in the diet of Limousin cattle was associated with a favorable increase in muscle protein content. This is particularly relevant given the growing global demand for high-quality animal protein, driven by increased meat production and consumption over the past five decades [61]. Intramuscular fat, commonly referred to as marbling, plays a critical role in determining meat tenderness and flavor [62]. In our study, the experimental groups from both breeds exhibited reduced intramuscular fat levels, potentially indicating lower tenderness and a less favorable sensory profile. However, leaner meat is often preferred by health-conscious consumers due to its lower caloric density and potential dietary benefits [56].

### 4.2. Fatty Acid Profile

The level and composition of fatty acids in meat significantly affect its nutritional quality, sensory attributes, and potential health implications for consumers [63]. SFAs primarily function as an energy source; however, excessive dietary intake is associated with elevated concentrations of atherogenic lipoproteins and increased blood coagulation, thereby contributing to cardiovascular risk [64]. The predominant SFAs found in ruminant-derived meat products include myristic acid (C14:0), palmitic acid (C16:0), and stearic acid (C18:0). Of these, C14:0 and C16:0 are particularly atherogenic, as they are known to raise blood cholesterol levels, which is an established risk factor for ischemic heart disease in humans [65]. In the present study, dietary supplementation with protected cod liver oil in Limousin bulls led to a reduction in the proportions of myristic acid (C14:0) and stearic acid (C18:0) in subcutaneous adipose tissue. These findings align with previous research showing that the inclusion of plant-derived oils rich in UFAs in ruminant diets can reduce SFA levels and concurrently increase the proportion of UFAs [57,59]. A similar trend was observed in the longissimus dorsi muscle, where cod liver oil supplementation in Limousin bulls resulted in a decrease in total SFA content. However, in subcutaneous fat, regardless of breed, the dietary inclusion of cod liver oil was associated with a reduction in the total level of UFA and a corresponding increase in total SFA content. These contrasting effects between muscle and fat tissues may reflect tissue-specific lipid metabolism and warrant further investigation.

Monounsaturated fatty acids are characterized by the presence of a single double bond in their carbon chain, and can occur in both cis and trans configurations. Among MUFAs, cis-oleic acid (C18:1n9c) represents the predominant form found in beef [65,66]. While beef meat naturally contains trans fatty acids, excessive dietary intake of these compounds has been associated with adverse health outcomes in humans. Specifically, trans fatty acids have been shown to increase low-density lipoprotein (LDL, “bad cholesterol”) and decrease high-density lipoprotein (HDL, “good cholesterol”), thereby elevating the risk of cardiovascular symptoms such as myocardial infarction and stroke [66,67]. As such, the UK Scientific Advisory Committee on Nutrition (SACN) recommends that trans fats contribute no more than 2% of total daily energy intake [68]. In the present study, supplementation with protected cod liver oil led to a favorable increase in cis-oleic acid and total MUFA levels in both the muscle and subcutaneous fat of Limousin and Red Angus cattle, without affecting the content of trans fatty acids. These findings contrast with those of Wistuba et al. (2007) [35], who reported a reduction in C18:1 cis and total MUFA levels in meat following dietary supplementation with 3% fish oil. Similarly, Scollan et al. (2001) [69] observed a decrease in C18:1n9 cis and a concurrent increase in C18:1 trans isomers when cattle were fed diets supplemented with unprotected fish oil and flaxseed. In both of those studies, the lipid supplements were not protected, which may account for the differences in fatty acid metabolism and deposition observed in the present study.

In addition to cis-MUFAs, PUFAs are essential for human health due to their regulatory role in lipid metabolism and their ability to reduce blood cholesterol and triglyceride levels [11,70]. However, elevated levels of PUFAs in meat may negatively impact sensory attributes, particularly by contributing to the development of undesirable flavors upon cooking [71]. According to literature data, dietary supplementation with fish oil increases the PUFA content in beef [35,72]. Meanwhile, Scollan et al. (2001) [69] reported that the inclusion of fish oil and flaxseed in cattle diets led to a reduction in arachidonic acid (C20:4n-6) and an increase in α-linolenic acid (C18:3n-3) in the longissimus thoracis muscle. In the present study, a reduction in both C20:4n-6 and C18:3n-3 fatty acid levels was observed in the meat of Red Angus cattle. However, no significant effect of the dietary supplementation was detected on the total PUFA content in either muscle or fat tissue in both breeds. As noted by Jeong et al. (2024) [70], excessive consumption of omega-6 fatty acids and a high dietary omega-6 to omega-3 (n-6/n-3) ratio are associated with an increased risk of cardiovascular disease, cancer, inflammatory disorders, and autoimmune conditions. The inclusion of flaxseed oil, both protected and unprotected, in bull diets has been shown to enhance the fatty acid profile of beef by increasing n-3 fatty acid content and improving the n-6/n-3 ratio [59]. Similarly, the use of fish oil in cattle diets has been reported to elevate n-3 fatty acid levels and improve the n-6/n-3 ratio [35,72]. In contrast, the present study found that fish oil supplementation resulted in an increased n-6/n-3 ratio in the meat of Limousin cattle, whereas a decrease in n-3 levels was observed in Red Angus cattle. Nevertheless, the observed n-6/n-3 ratios and n-3 fatty acid levels remained within the typical range for beef and lean meat, which are considered nutritionally desirable components of the human diet [69,73].

In the present study, breed was found to influence the fatty acid composition of muscle tissue. Meat from Red Angus cattle exhibited higher levels of SFAs and MUFAs, whereas Limousin meat was characterized by higher levels of PUFAs, a more favorable PUFA/SFA ratio, and elevated concentrations of both n-3 and n-6 fatty acids. No statistically significant breed-related differences were observed in the fatty acid composition of subcutaneous adipose tissue. The findings of this study are in agreement with available literature. Prieto et al. (2011) [18] reported higher concentrations of SFAs, MUFAs, and n-3 fatty acids in the meat of Aberdeen Angus cattle compared to Limousin. Conversely, Jukna et al. (2013) [14] observed that Limousin beef contained higher levels of PUFAs, trans fatty acids, and n-6 fatty acids, along with an elevated n-6/n-3 ratio, but lower concentrations of MUFAs and n-3 fatty acids when compared to Angus meat. In general, the PUFA/SFA ratio in beef is relatively low, typically around 0.1, with the exception of double-muscling breeds, which tend to be very lean and may exhibit PUFA/SFA ratios ranging from 0.5 to 0.7 [74]. For optimal cardiovascular health, the World Health Organization (2003 and 2023) [9,10] recommends a PUFA/SFA ratio greater than 0.45 in the human diet. In the current study, a relatively high PUFA/SFA ratio was recorded in the meat of Limousin cattle, suggesting its potential nutritional advantage for health-conscious consumers.

The short-term use of feed additives, including oils, in the diet may be an attractive solution for the meat industry due to economic considerations [43]. Within a short period, it is possible to obtain meat with desirable properties [43,45]. The use of additives derived from oilseed plants and oils is considered safe. Cancino-Padilla et al. (2021) [75] demonstrated that supplementation with 3% lipids in dry matter does not negatively affect the rumen microbiome. According to INRA guidelines (2007) [46], the fat content in the dry matter of feed intended for finishing cattle should not exceed 6%. Fish oils and oils derived from oilseed crops contain long-chain unsaturated fatty acids, including omega-3 (n-3) fatty acids [31]. In ruminants, these unsaturated fatty acids undergo physiological biohydrogenation in the rumen, resulting in the formation of saturated fatty acids [76]. To prevent this transformation and preserve the biological value of the supplemented fats, unsaturated fatty acids are often administered in rumen-protected forms, which have been shown to improve the fatty acid profile of beef [31,77]. In the present study, cod liver oil was used as a dietary supplement to enhance fat quality. One of the key factors influencing the fatty acid composition of both subcutaneous and intramuscular fat is cattle breed [19]. The fatty acid profile of beef differs between Limousin and Red Angus cattle. Red Angus beef typically exhibits higher concentrations of saturated fatty acids (SFAs), particularly stearic acid, whereas Limousin beef is characterized by a greater proportion of polyunsaturated fatty acids (PUFAs) [18,78]. Bovine adipose tissue contains approximately 39.3 kJ/g of energy. As a result, animals with greater fat deposition require more dietary energy, which increases feed conversion efficiency [79]. This may explain the higher levels of SFAs, and the lower levels of PUFAs and n-3 fatty acids observed in Red Angus meat compared to Limousin meat in the present study. Additionally, due to the higher fat content of Red Angus beef, a longer period of cod liver oil supplementation may be necessary to observe comparable improvements in fatty acid composition. This hypothesis requires further investigation. Intramuscular fat is in the perimysial spaces between muscle fibers, and its accumulation contributes to visible marbling [80]. Marbling is considered one of the most important factors influencing beef quality and sensory characteristics [20]. Theoretically, Red Angus beef should exhibit a higher degree of marbling compared to Limousin beef. The present study also revealed differences in the response of subcutaneous and intramuscular fat to cod liver oil supplementation. These differences may be attributed to genetic factors. In Angus and Limousin cattle, the genetic correlation for intramuscular fat deposition is r = 0.32, whereas for subcutaneous fat, it is only r = 0.05 [81]. In cattle, subcutaneous fat tends to accumulate more rapidly than intramuscular fat, which may also influence its fatty acid profile. Subcutaneous fat typically contains a higher proportion of saturated fatty acids than intramuscular fat [76]. The fatty acid composition influences key physical properties such as melting point, consistency, and firmness of both fat and meat. Adipocytes with a higher proportion of saturated fatty acids are firmer and lighter in color. In contrast, unsaturated fatty acids are more prone to oxidation, particularly those containing more than two double bonds, which may lead to rancidity and discoloration of the meat [17,82]. An elevated PUFA content increases susceptibility to lipid oxidation [83]. However, it is important to note that thermal oxidation of unsaturated fatty acids contributes to desirable flavor and aroma in cooked meat, whereas autoxidation leads to undesirable off-flavors [75,83]. Therefore, meat from Limousin cattle supplemented with cod liver oil may require protection against autoxidation, while Red Angus meat is more likely to retain its sensory properties for a longer period. Further studies are needed to evaluate both the health benefits and the sensory quality of beef from animals receiving cod liver oil supplementation.

The atherogenic index (AI) reflects the relationship between hypercholesterolemic fatty acids and those considered protective, which inhibit the formation of atherosclerotic plaques and contribute to the reduction in plasma cholesterol levels, thereby supporting the prevention of coronary heart disease [84]. The thrombogenic index (TI) is defined as the ratio of pro-thrombogenic (primarily saturated) to anti-thrombogenic fatty acids, and it serves as an indicator of the potential for thrombus (blood clot) formation within blood vessels. From a human health perspective, it is generally recommended that the TI value remain as low as possible [85]. Both AI and TI values have been reported to be more strongly influenced by species-specific characteristics than by production systems or feeding regimes [86]. In the present study, a reduction in TI was observed in subcutaneous fat tissue across both breeds in response to cod liver oil supplementation. Furthermore, Limousin cattle exhibited lower AI and TI values compared to Red Angus cattle, suggesting a more favorable fatty acid profile in terms of human cardiovascular health.

## 5. Conclusions

In summary, short-term supplementation with protected cod liver oil led to a reduction in intramuscular fat content, and altered the fatty acid composition of both muscle and subcutaneous fat tissues. Meat and fat from both Limousin and Red Angus cattle exhibited a desirable increase in cis-oleic acid, without affecting the trans fatty acid content. In contrast, in the adipose tissue of both breeds, higher overall levels of MUFAs were observed in the group receiving cod liver oil. Given that myristic acid (C14:0) and stearic acid (C18:0) in the human diet are associated with adverse health effects, the inclusion of cod liver oil in the diet of beef cattle may be considered beneficial for improving the quality of subcutaneous fat. In Limousin cattle, supplementation resulted in a reduced proportion of both C14:0 and C18:0 fatty acids in subcutaneous adipose tissue, whereas in Red Angus bulls, a reduction was observed specifically in C18:0. The inclusion of protected cod liver oil in the finishing diet contributed to the production of beef with health-promoting characteristics, supporting its suitability for inclusion in a balanced human diet. However, long-term studies are required to evaluate the sustainability of human dietary use and its potential impact on human health.

## Figures and Tables

**Table 1:** Chemical analysis of the composition of feeds and the complete feed mixture for fattening cattle included in the rations.

Item	Concentration				
Ingredient (% of DM)					
Triticale	37.3				
Rapeseed cake	29				
Wheat	19				
Protein concentrates for fattening cattle	6				
Soybean meal	4				
Sodium bicarbonate	3				
Soybean oil	1				
Vitamin and mineral mix	0.7				
	DM	CP	EE	NDF	ADF
	(%)	(% of DM)			
Proximate composition	88.4	23.9	2.9	12.8	6.9
Corn silage	38.57	2.83	1.08	16.5	8.09
Grass silage	37.00	9.19	1.24	22.39	12.23
Barley straw	88.86	4.12	2.69	71.79	49.45

DM = dry matter; CP = crude protein; EE = ether extract; NDF = neutral detergent fiber; ADF = acid detergent fiber.

**Table 2:** Fatty acid composition of cod liver oil.

Fatty Acid	g/100 g Fat	Fatty Acid	g/100 g Fat
C14:0	3.64	C20:5	8.22
C16:0	11.60	C22:0	0.70
C16:1	9.11	C22:1	10.01
C18:0	2.22	C22:5	1.86
C18:1	24.50	C22:6	11.31
C18:2	2.36	n-3	22.47
C18:3	1.08	n-6	2.36
C20:1	10.01	n-6/n-3	0.11

**Table 3:** Fat and protein content in muscle tissue (%) in beef cattle.

	Limousine		Red Angus		SEM	*p*-Value
	CL	EL	CA	EA		
Protein	20.81 ^Bb^	22.65 ^A^	22.45 ^A^	21.85 ^a^	0.178	0.000
Fat	0.74 ^Ba^	0.57 ^Bb^	1.21 ^Aa^	1.03 ^Ab^	0.068	0.003

Means within the same row followed by different superscript letters differ significantly: lowercase letters (a, b) indicate significance at *p* < 0.05; uppercase letters (A, B) indicate significance at *p* < 0.01. CL—control group, Limousin bulls; EL—experimental group, Limousin bulls; CA—control group, Red Angus bulls; EA—experimental group, Red Angus bulls. SEM—standard error of the mean.

**Table 4:** Concentration of saturated fatty acids in muscle tissue (g/100 g fat) in beef cattle.

Saturated Fatty Acid	Limousine		Red Angus		SEM	*p*-Value
	CL	EL	CA	EA		
C_14:0_	1.33 ^B^	1.08 ^Bb^	1.71 ^a^	2.00 ^A^	0.009	0.010
C_15:0_	0.55 ^A^	0.40 ^B^	0.42 ^B^	0.42 ^B^	0.019	0.007
C_16:0_	20.21 ^B^	19.42 ^B^	22.87 ^A^	23.38 ^A^	0.437	0.001
C_17:0_	0.92	0.81	0.88	0.98	0.033	0.302
C_18:0_	19.70	18.27	19.21	19.66	0.442	0.589
C_20:0_	0.25 ^a^	0.24 ^a^	0.17 ^b^	0.17 ^b^	0.013	0.014
∑SFA	42.69 ^A^	39.99 ^B^	45.22 ^A^	46.60 ^A^	0.478	0.000

CL—control group, Limousin bulls; EL—experimental group, Limousin bulls; CA—control group, Red Angus bulls; EA—experimental group, Red Angus bulls. SFA—saturated fatty acid. SEM—standard error of the mean. Means within the same row followed by different superscript letters differ significantly: lowercase letters (a, b) indicate significance at *p* < 0.05; uppercase letters (A, B) indicate significance at *p* < 0.01.

**Table 5:** Proportion of unsaturated fatty acids, atherogenic index, and thrombogenic index in muscle tissue (g/100 g fat) in beef cattle.

Unsaturated Fatty Acid	Limousine		Red Angus		SEM	*p*-Value
	CL	EL	CA	EA		
C_14:1_	0.40	0.62	0.45	0.57	0.077	0.344
C_15:1_	0.35	0.29	0.32	0.34	0.010	0.193
C_16:1_	2.19 ^B^	2.11 ^B^	2.94 ^A^	3.20 ^A^	0.120	0.000
C_17:1_	0.76 ^b^	0.63 ^Bb^	0.84 ^a^	0.99 ^A^	0.039	0.002
C_18:1n9c_	26.11 ^B^	23.85 ^B^	31.44 ^Ab^	34.87 ^Aa^	0.951	0.000
C_18:1n8c_	1.92 ^b^	2.23 ^a^	1.96 ^b^	1.92 ^b^	0.049	0.030
C_18:1n9t_	0.40	0.42	0.28	0.29	0.038	0.331
C_18:1n7t_	1.25	1.19	1.11	1.56	0.067	0.108
C_20:1_	0.12 ^Bb^	0.19 ^b^	0.30 ^Aa^	0.28	0.019	0.002
MUFA	33.42 ^B^	30.78 ^B^	39.91 ^Aa^	44.47 ^Ab^	1.190	0.000
C_18:2n6c_	10.96 ^A^	15.38 ^A^	6.87 ^B^	4.32 ^B^	1.046	0.000
C_18:2n6_	1.50	0.31	0.33	0.34	0.020	0.864
C_18:3n3_	2.36 ^A^	2.61 ^A^	1.53 ^Ba^	0.94 ^Bb^	0.153	0.000
C_20:4n6_	4.24 ^A^	5.17 ^A^	2.60 ^ab^	1.15 ^Bb^	0.434	0.001
C_20:5n3_	1.00 ^A^	1.06 ^Aa^	0.56 ^b^	0.26 ^B^	0.087	0.001
C_22:6 n3_	0.27 ^A^	0.30 ^A^	0.12 ^B^	0.06 ^B^	0.032	0.003
PUFA	19.65 ^A^	24.55 ^A^	11.92 ^B^	6.98 ^B^	1.674	0.000
∑ UFA	53.07	55.2	51.77	51.43	0.6467	0.116
PUFA/∑SFA	0.50 ^A^	0.63 ^A^	0.27 ^B^	0.15 ^B^	0.049	0.000
n3	3.45 ^A^	3.80 ^A^	2.17 ^Ba^	1.17 ^Bb^	0.248	0.000
n6	16.20 ^A^	20.75 ^A^	9.75 ^B^	5.80 ^B^	1.442	0.000
n3/n6	0.21	0.19	0.23	0.20	0.006	0.230
n6/n3	4.76 ^b^	5.44 ^a^	4.41	5.03	0.092	0.017
AI index	0.49 ^b^	0.43 ^B^	0.58 ^A^	0.61 ^Aa^	0.020	0.001
TI index	1.23 ^B^	1.05 ^B^	1.40 ^C^	1.57 ^A^	0.0543	0.000

CL—control group, Limousin bulls; EL—experimental group, Limousin bulls; CA—control group, Red Angus bulls; EA—experimental group, Red Angus bulls. SFA—saturated fatty acid. MUFA—monounsaturated fatty acid; PUFA—polyunsaturated fatty acid; UFA—unsaturated fatty acid (MUFA + PUFA); AI—atherogenic index; TI—thrombogenic index. SEM—standard error of the mean. Means within the same row followed by different superscript letters differ significantly: lowercase letters (a, b) indicate significance at *p* < 0.05; uppercase letters (A, B) indicate significance at *p* < 0.01.

**Table 6:** Proportion of saturated fatty acids in subcutaneous adipose tissue (g/100 g fat) in beef cattle.

Saturated Fatty Acid	Limousine		Red Angus		SEM	*p*-Value
	CL	EL	CA	EA		
C_14:0_	2.23 ^Bb^	2.55 ^a^	2.70 ^A^	2.88 ^A^	0.066	0.003
C_15:0_	0.87	0.88	0.76	0.69	0.030	0.091
C_16:0_	19.98 ^B^	19.38 ^B^	22.49 ^A^	23.87 ^A^	0.472	0.000
C_17:0_	1.55 ^Aa^	1.42 ^b^	1.42 ^b^	1.30 ^Ba^	0.025	0.002
C_18:0_	39.57 ^Aa^	35.64 ^b^	36.11 ^b^	28.62 ^Ba^	0.921	0.000
C_20:0_	0.53 ^Aa^	0.61 ^a^	0.47	0.36 ^Bb^	0.028	0.008
∑SFA	64.71 ^A^	60.44 ^BCa^	63.93 ^Cb^	57.38 ^Bb^	0.675	0.000

CL—control group, Limousin bulls; EL—experimental group, Limousin bulls; CA—control group, Red Angus bulls; EA—experimental group, Red Angus bulls. SFA—saturated fatty acid. SEM—standard error of the mean. Means within the same row followed by different superscript letters differ significantly: lowercase letters (a, b) indicate significance at *p* < 0.05; uppercase letters (A, B) indicate significance at *p* < 0.01.

**Table 7:** Proportion of unsaturated fatty acids in subcutaneous adipose tissue (g/100 g fat) in beef cattle.

Unsaturated Fatty Acid	Limousine		Red Angus		SEM	*p*-Value
	CL	EL	CA	EA		
C_14:1_	0.63	0.75	0.64	0.69	0.021	0.151
C_15:1_	0.66	0.68	0.61	0.58	0.018	0.149
C_16:1_	2.09 ^Bb^	2.57 ^a^	2.41	2.75 ^A^	0.072	0.039
C_17:1_	0.53 ^b^	0.53 ^b^	0.59 ^b^	0.68 ^Aa^	0.019	0.004
C_18:1n9c_	20.12 ^B^	22.64 ^B^	22.41 ^B^	28.20 ^A^	0.708	0.001
C_18:1n8c_	1.23 ^b^	1.38 ^a^	1.17 ^b^	1.43 ^a^	0.037	0.017
C_18:1n9t_	0.51	0.61	0.39	0.44	0.044	0.403
C_18:1n7t_	2.63	3.12 ^a^	2.32 ^b^	2.71	0.094	0.034
C_20:1_	0.36	0.36	0.35	0.30	0.041	0.632
MUFA	28.63 ^Bb^	32.34 ^a^	30.66 ^a^	36.29 ^Ab^	0.678	0.001
C_18:2n6c_	1.45	1.67	1.21	1.47	0.049	0.598
C_18:2n6_	0.25	0.26	0.27	0.34	0.016	0.247
C_18:3n3_	0.53	0.52	0.46	0.51	0.018	0.971
C_20:4n6_	0.51	0.71	0.38	1.78	0.424	0.772
C_20:5n3_	0.62	0.48	0.38	0.59	0.786	0.371
PUFA	2.99	3.53	2.34	3.29	0.357	0.714
∑ UFA	31.62 ^B^	36.87 ^A^	33.49 ^B^	40.37 ^A^	0.758	0.001
PUFA/∑SFA	0.04	0.06	0.04	0.06	0.006	0.622
n3	0.89	1.35	0.64	0.52	0.280	0.648
n6	1.85	2.18	1.70	2.77	0.949	0.486
n3/n6	0.48	0.51	0.41	0.28	0.087	0.771
n6/n3	2.64	3.56	1.85	3.23	0.283	0.532
AI index	0.93 ^a^	0.53 ^b^	1.01 ^b^	0.9	0.194	0.010
TI index	3.41 ^Aa^	2.85 ^b^	3.35 ^a^	2.61 ^Bb^	0.104	0.007

CL—control group, Limousin bulls; EL—experimental group, Limousin bulls; CA—control group, Red Angus bulls; EA—experimental group, Red Angus bulls. SFA—saturated fatty acid. MUFA—monounsaturated fatty acid; PUFA—polyunsaturated fatty acid; UFA—unsaturated fatty acid (MUFA + PUFA); AI—atherogenic index; TI—thrombogenic index. SEM—standard error of the mean. Means within the same row followed by different superscript letters differ significantly: lowercase letters (a, b) indicate significance at *p* < 0.05; uppercase letters (A, B) indicate significance at *p* < 0.01.

## Data Availability

Data are contained within the article.
